# Effect of Buttermilk on the Physicochemical, Rheological, and Sensory Qualities of Pan and Pita Bread

**DOI:** 10.1155/2017/2054252

**Published:** 2017-11-26

**Authors:** Amani H. Al-Jahani

**Affiliations:** Nutrition and Food Science Department, College of Home Economics, Princess Nourah Bint Abdulrahman University, Riyadh, Saudi Arabia

## Abstract

The aim of this study was to evaluate the influence of buttermilk on the physicochemical and sensory attributes of pan and pita breads. Different amounts of buttermilk (30, 60, and 100% of added water) were mixed with other ingredients of pan and pita bread formulations. The doughs and bread were analyzed for rheological, physicochemical, and sensory qualities. The results demonstrated that incorporation of different concentrations of buttermilk in bread formulations progressively enhanced water absorption capacity, dough development time, gelatinization temperature, and peak viscosity, whereas it reduced the dough stability and temperature at peak viscosity. Supplementation of wheat flour with 30% buttermilk significantly (*P* ≤ 0.05) enhanced the physical properties of pan bread compared to nonsupplemented control. Incorporation of different percentages of buttermilk in bread formulation concomitantly (*P* ≤ 0.05) increased protein, oil, and ash contents and it reduced the carbohydrate contents of both types of bread. Incorporation of 60 and 100% of buttermilk in bread formula showed low scores of all sensory attributes compared to control and 30% buttermilk containing pan and pita bread. In conclusion, supplementation of bread formulas with 30% buttermilk is recommended for improving the nutritional and sensorial qualities of pan and pita bread.

## 1. Introduction

Wheat is an important and most widely cultivated crop in the world, and wheat-based products provide 20% of all calories consumed by people around the globe. The most important application of wheat is in bread making processes where wheat flour represents the main ingredient. Throughout history, bread is one of the oldest and most staple foods prepared and consumed by humans throughout the world [1]. In recent decades, the global production and consumption of bread have considerably increased due to population explosion and changes in lifestyle and eating habits [2]. In Middle East countries, particularly in Saudi Arabia, the major types of bread prepared and consumed are pan (Samouli) and pita (Mafrood) bread and these bread types are mostly made from white flour [3]. However, during wheat milling processes most of the important nutrients are removed resulting in flour with low protein content and quality due to the deficiency of wheat proteins lacking essential amino acids such as lysine and threonine in wheat protein [4]. In addition, removing the wheat bran and germ also may result in losses of many nutritious and health promoting compounds such as dietary fiber, minerals, vitamins, and various antioxidant compounds [5]. To overcome such limitations, supplementation of wheat flour with protein rich and nutritious sources has become an increasingly important and challenging research field in recent years. In this regard, several research reports on supplementation or fortification of bread wheat flour with grains and legumes flour have been published [6–12].

Buttermilk, a low-fat milky liquid leftover after the churning of cream, is one of the most important functional dairy products that have excellent health and disease curing potentials and it is now receiving high interest from consumers all over the world. In addition, buttermilk is also considered as an excellent source of nutritional elements such as minerals (potassium, phosphorus, and calcium), vitamin B12, riboflavin, enzymes, and protein [13]. Moreover, buttermilk has a fresh and piquant taste and has applications in a wide variety of foods such as refreshing drinks, low fat yogurt, cheese, ice cream, nutritious bakery products, and confectionaries [14]. Furthermore, buttermilk has several therapeutic potentials such as cholesterol reduction, blood pressure reduction, antiviral effects, and anticancer effects [15–17]. In baking industry, buttermilk could be used to enhance the rheological, nutritional, and organoleptic properties of bread [18, 19]. Due to its high nutritional and health promoting potentials, utilization of buttermilk as a supplement in bread formulations received significant attention in recent years [18, 19]. Therefore, the main aims of the present study are to utilize buttermilk in pan and pita bread formulations and evaluate the influence of buttermilk on the physicochemical and sensory attributes of pan and pita breads.

## 2. Materials and Methods

### 2.1. Materials

Hard wheat flour (75% extraction) was obtained from Al-Andalus milling factory, Riyadh, Saudi Arabia. Margarine of hydrated soybean and cotton seed oils was purchased from Goody Middle East Company, Dubai, United Arab Emirates. BakeMate bread improver (containing *α*-amylase) was brought from Ahmad Abid trading company, Riyadh, Saudi Arabia. Sugar and salt were obtained from local markets in Riyadh, Saudi Arabia. Fresh buttermilk was obtained from Almarai Company, Saudi Arabia. All chemicals used are of analytical grade.

### 2.2. Preparation of Pan Bread (Loaf)

The dough was prepared following the standard straight dough method 10-10A [20] with some modifications. Briefly, the ingredients (Table 1) were mixed on the basis of 1000 g wheat flour (14% moisture content) to the levels of 3% instant yeast, 3% margarine, 2% salt, 5% sugar, and 0.01% improver. Then, different amounts water and buttermilk (30, 60, and 100% of the added water) were added based on the flour optimum absorption as determined by farinograph. After that, all contents were thoroughly mixed (Tyrone, model TR 202, UK) to the optimum dough development time as determined by farinograph. Then, the dough was formed in ball shape and kept in a fermentation tank for 30 min at 32°C and 85% relative humidity. Thereafter, the dough was freed from gases and divided into three portions of 550 g each and then fermented again for 20 min under the above conditions. Then, the fermented dough was passed through bread forming machine and put into baking pans (22 × 6 × 7 cm) and kept again in the fermentation tank for final fermentation for 30 min at the same condition. After fermentation, the dough was baked in the electric rotary oven (National Co. Ltd., Kyoto, Japan) for 20 min at 225°C. After cooling for 30 min at room temperature, the bread volume was estimated by the seed displacement method. The volume of seeds displaced by the bread was considered as the bread volume. The specific volume of the bread was calculated according to the AACC method [20] by dividing volume (CC) by weight (g).

### 2.3. Preparation of Pita Bread (Flat)

The method of preparation of Arabian bread was used to prepare pita bread as described elsewhere [21]. Briefly, the ingredients (Table 1) were mixed on the basis of 1000 g wheat flour (14% moisture content) to the levels of 3% instant yeast, 5% sugar, 1% salt, and 0.01% improver. Then, different amounts of water and buttermilk (30, 60, and 100% of the added water) were added based on the flour optimum absorption as determined by farinograph. The contents were mixed to form dough and fermented as described for pan bread. Thereafter, the dough was divided into portions (150 g each) and formed into balls by hand and then fermented again for 20 min under the above-mentioned conditions. Then the flat bread was created by using wooden cylinder having the diameter of 20 cm and thickness of 6 mm and left for final fermentation for 30 min at the same conditions. The bread was baked at 350°C for 2 min and then left to cool for 30 min before analysis.

### 2.4. Approximate Composition

The approximate composition of bread samples was determined following the official standard method [22]. Moisture content was measured using oven drying method and weight measurements before and after drying (AOAC 935.29). Protein was determined by Kjeldahl method (AOAC 988.05). Oil and ash were estimated by Soxhlet method (AOAC 963.15) and drying methods (AOAC 942.05), respectively. Carbohydrate was calculated by differences.

### 2.5. Determination of Dough Rheology

The rheological properties of wheat dough supplemented with different concentrations of buttermilk (30, 60, and 100% of added water) followed the standard methods of American Association of Cereal Chemists [20]. Farinogragh and amylograph measurements were carried out according to AACC 54-21 and 54-10 methods, respectively, using Barbender instruments (C.W. Brabender Instrument Inc., South Hackensack, NJ, USA).

### 2.6. Sensory Attributes

Sensory analysis of bread samples was performed immediately after cooking by 10 semitrained panelists (male, age range 20–35 years old). The panelists were initially trained for sensory evaluation, and the assessment was carried out in three independent sessions. The sensory quality of pan bread was assessed using a 10-point hedonic scale (10 = like extremely, 1 = dislike extremely) and panelists were asked to assess both external and internal properties of the bread such as aroma, taste, crumb texture, crumb color, crumb cell uniformity, and general acceptability, whereas for pita bread a 5-point hedonic scale (5 = like, 1 = dislike) was used for the external (color of the loaf, the shape and degree of consistency, and the degree of cracking and breaking) and internal (color of the pulp, texture of the pulp, the smell and the taste, and the degree of symmetry of the top layer with the bottom layer) features. All samples were coded with three-digit random numbers and served randomly to the assessors. Sensory analysis was performed in three sessions, and the mean values of the scores of 10 panelists for each sample and session were calculated and used in data analysis.

### 2.7. Statistical Analysis

Triplicate experiments and measurements were carried out and the data collected were subjected to statistical analysis using SAS program (SAS 8.0 software, SAS Institute, Inc., Cary, NC, USA). Analysis of variance (ANOVA) and Duncan's Multiple Range Test were performed to analyze the effect of treatments on the chemical, rheological, and sensory characteristics of the pan and pita bread. Data were presented as mean, and standard deviation (SD) and statistical significance were accepted at the probability of *P* ≤ 0.05.

## 3. Results and Discussion

### 3.1. Dough Rheology

The dough rheological properties of wheat flour supplemented with different concentrations of buttermilk were analyzed by farinograph and amylograph. The farinograph results indicated that supplementation of wheat flour with buttermilk significantly affected the water absorption, dough development time, dough stability, and degree of softening (Table 2). Increasing the concentrations of buttermilk in the dough progressively (*P* ≤ 0.05) increased the water absorption from 60% in control to 67% in wheat flour supplemented with 100% buttermilk. This could be due to the increase of the protein solubility and content of the dough following the addition of buttermilk whose protein is characterized by its high solubility, hydrophobicity, and absorption capacity. Furthermore, added buttermilk could result in a structural modification in the dough which may allow absorption of more water due to hydrogen bonding. High water absorption capacity of dough represents consistency which is one of the appealing characteristics in bread making. The quantity of added water is considered to be very important for the distribution of the dough materials, their hydration, and the gluten protein network development. Similar to our observations, Hassan et al. [18] reported that replacing water with different concentrations of buttermilk in bread formulation substantially increased water absorption of the dough in a concentration dependent manner. In addition, several research reports have demonstrated that supplementation of wheat flour with vegetable and legumes flour, dairy products, or protein isolates significantly increased the water absorption capacity [7, 9, 23, 24]. By contrast, Madenci and Bilgiçli [19] reported that addition of dairy by-products (whey protein concentrate and buttermilk powder) to wheat flour reduced the water absorption of the formed dough. Our results indicated that dough development time was also increased (*P* ≤ 0.05) with an increase in buttermilk concentration to 30 and 60% and then decreased again at 100% buttermilk. The enhancement of dough development time upon addition of buttermilk could be due to the differences in the physicochemical properties of the constituents of the buttermilk and those of the wheat flour. Similarly, Hassan et al. [18] and Bilgin et al. [25] stated that the development time of wheat dough was significantly increased upon replacing water with different concentrations of fermented skimmed milk, acid whey, and buttermilk. In addition, Madenci and Bilgiçli [19] reported that incorporation of dairy by-products in wheat dough significantly enhanced the dough development time. Moreover, Mohammed et al. [9] indicated that addition of chickpea flour to wheat flour increased the dough development time. Our findings also showed that incorporation of buttermilk in wheat dough decreased the dough stability compared to control and the reduction was concomitant with an increase in the concentration of buttermilk in the dough. The reduction could be due to the fact that added buttermilk constituents could disrupt the wheat gluten-starch network, competing with wheat flour proteins for water and likely the proteolytic activity in the buttermilk hydrolyze wheat gluten and then decreased its stability. Similarly, Hassan et al. [18] reported a reduction in dough stability at higher supplementation levels of acid whey, buttermilk, and skimmed milk powder in pan bread formulations. In addition, a similar decrease in dough stability was also observed by Sabanis and Tzia [11], Anton et al. [26], Gadallah et al. [27], and Pasha et al. [28] as the percentage level of legume flour in the blend increased. Our results also revealed that the effects of buttermilk fortification on the departure time and degree of softening were minor. Overall, the farinograph analysis demonstrated that incorporation of different concentration of buttermilk in bread formulations positively affected the water absorption capacity and development time of dough, whereas it showed an adverse impact on the dough stability.

The amylograph results (Table 3) showed that fortification of wheat flour with buttermilk significantly (*P* ≤ 0.05) affected the gelatinization temperature, peak viscosity, and temperature at peak viscosity of fortified dough compared to control samples. Gelatinization temperature and peak viscosity concomitantly (*P* ≤ 0.05) increased with increase in the concentration of buttermilk in the dough, while the temperature at peak viscosity showed concomitant reduction as the buttermilk level increased. The increase in the peak viscosity and gelatinization temperature could be attributed to the added lactose sugar from buttermilk. Similarly, it has been reported that addition of mushroom powder to wheat flour substantially increased peak viscosity from 820 to 1700 BU [29]. Furthermore, increases in peak viscosity and gelatinization temperature were observed in composite flours of wheat, cereals, legumes, or sago flours [30–33]. By contrast, other reports have indicated that addition of legumes protein isolates and milk proteins reduced the peak viscosity of the dough [34, 35]. The difference could be attributed to the variation in the added materials that in the latter is protein isolates which are devoid of sugars or starches whereas in the former the whole materials are added which might contains sugars and starches that increase the viscosity and gelatinization temperature.

### 3.2. Physical Properties of Pan and Pita Bread

**Figure 1:**
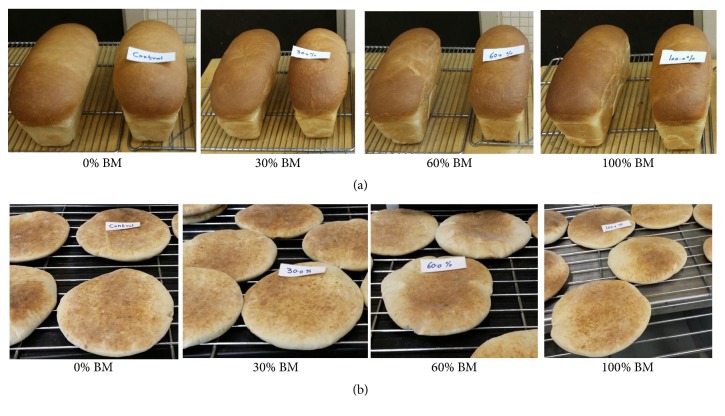
Physical appearance of pan (a) and pita (b) breads containing different levels of buttermilk (BM). Control; 0% BM, 30% butter milk; 30% BM, 60% buttermilk; 60% BM, and 100% buttermilk; 100% BM.

Wheat flour with or without buttermilk supplementation was used for the preparation of two types of bread frequently consumed in Kingdom of Saudi Arabia. The results of the physical properties of pan bread are shown in Table 4 and Figure 1(a). The results indicated that supplementation of wheat flour with buttermilk significantly (*P* ≤ 0.05) enhanced the weight and volume of pan bread compared to nonsupplemented control. Strikingly, loaf weight and volume enhancement during baking of pan bread containing buttermilk is a desirable quality attribute as consumers are often attracted to bread with high weight and volume believing that it has more substance for the same price. The loaf weight concomitantly (*P* ≤ 0.05) increased with increase in the concentration of buttermilk reaching the maximum at 100% buttermilk supplementation. Increase in bread weight may be due to increased water absorption capacity of buttermilk containing dough (Table 2). The highest (*P* ≤ 0.05) loaf volume (2883.33 ± 28.86 cm) and loaf specific volume (5.92 ± 0.07 cm^2^/g) were observed at 30% buttermilk supplementation followed by control (0% buttermilk), 60% and 100% buttermilk. Although they reduced by buttermilk supplementation at high percentages (60 and 100% buttermilk), the values of the loaf bread specific volume are still within the range 3.5–6.0 cm^2^/g that characterize the regular bread as identified by grain products research institutes [36]. The decrease (*P* ≤ 0.05) in loaf volume and specific volume at 60 and 100% buttermilk of the bread may be attributed to the reduction in the wheat structure forming proteins and a low ability of the dough to entrap air. In addition, the higher resistance of dough observed during dough handling and preparation might affect the gas retention in the dough and bread during baking and hence reduce the volume of the loaves. Moktan and Ojha [12] reported that increase in the percentage of germinated horse gram decreased the loaf volume and specific volume of bread but increased the loaf weight. Gani et al. [34] stated that addition of whey proteins, whey protein hydrolysates, casein, and casein hydrolysates significantly reduced the loaf specific volume.

On the other hand, the physical properties of pita bread also improved following the supplementation of wheat flour with different percentages of buttermilk (Figure 1(b)). Madenci and Bilgiçli [19] studied the effects of supplementation of wheat flour with whey protein concentrate powder and buttermilk powder on the quality of flat bread. They found that incorporation of buttermilk at 8% decreased the thickness and increased diameter and spread ratio of flat bread compared to controls. In addition, Madenci et al. [37] reported a decrement in thickness of lavash and an increment in diameter/spread of lavash with the usage of whey protein concentrate and buttermilk in the formulation. Overall, our results demonstrated that incorporation of buttermilk at 30% and above in bread mixture could improve the physical properties of the bread and thus it is well recommended to incorporate buttermilk in pan and pita bread formulations.

### 3.3. Chemical Composition of Pan and Pita Bread

The chemical composition of pan and pita bread made from wheat flour supplemented with different concentrations of buttermilk (0, 30, 60, and 100% of added water) is shown in Table 5. Generally, incorporation of buttermilk in bread formulation increased protein, oil, and ash contents and reduced the carbohydrate contents of both types of bread. The increases in protein, fat, and ash contents might result from the added buttermilk that contains appreciable amounts of these constituents, whereas the proportional reduction in carbohydrate is expected as the carbohydrate is calculated by subtracting protein, oil, and ash contents from 100%. The highest protein, fat, and ash contents of both types of bread were observed at 100% buttermilk supplementation while the lowest values were found in control samples suggesting concentration dependent effects of buttermilk on these constituents. In agreement with our findings, numerous reports indicated that supplementation of wheat flour with various legume flours or whey protein concentrate powder and buttermilk powder has concomitantly increased protein, oil, and ash contents and reduced carbohydrate content of fortified pan and flat breads [12, 18, 19, 38]. Overall, improvement of protein content in pan and pita bread following buttermilk supplementation could be of nutritional importance as the protein of animal sources in known by its good nutritional quality compared to cereal proteins. Thus, supplementation of bread with buttermilk could potentially improve the nutritional quality of pan and pita breads, those regularly consumed by people in Saudi Arabia, and hence could improve the nutritional and health status of humans.

### 3.4. Sensory Attributes of Pan and Pita Bread

Sensory assessment is a vital measure for quality assessment in a newly developed food product to attract consumers and to meet their requirements [29]. The choice of a food product depends on various factors such as character, mood and experience, and attitudes like sensory properties, health and nutrition, and price and value [8]. The sensory attributes of pan and pita bread prepared from wheat flour supplemented with or without different concentrations of buttermilk are presented in Table 6. For pan bread, the surface and crumb color were not affected by incorporation of buttermilk in the bread formulation. This is opposite to the findings of Gani et al. [34] where the color difference breads supplemented with milk protein concentrates and hydrolysates increased significantly with increasing levels of concentrates and hydrolysates; this was attributed to higher degree of Maillard browning which is influenced by the distribution of water and the reaction of reducing sugars and amino acids with increasing levels of concentrates and hydrolysates.* This study may* be attributed to the low concentrates of protein in breads supplemented of buttermilk which were less than the Gani et al. [34] study. However, inclusion of 60 and 100% of buttermilk significantly (*P* ≤ 0.05) reduced the degree of symmetry, the degree of cracking, and crumb texture compared to untreated control and that supplemented with 30% buttermilk. The overall evaluation also indicated that 30% buttermilk and control pan bread are superior to 60% and 100% buttermilk containing samples. Although it is not significant, 30% buttermilk containing pan bread outscores the control and those formulated with 60 and 100% buttermilk in all sensory attributes. These findings demonstrated that incorporation of buttermilk in pan bread formulations at 30% replacement of added water could enhance the sensory characteristics of pan bread.

For pita bread, the addition of buttermilk affected most of the sensory attributes with the exception of degree of cracking that was not significantly affected by incorporation of buttermilk. Both control and 30% buttermilk containing pita bread showed the highest scores of all sensory attributes compared to that containing 60 and 100% buttermilk. Similar to that of pan bread, the overall acceptability of pita bread was observed in control and 30% buttermilk containing pita bread. These findings suggested that incorporation of buttermilk in pita bread at a concentration of 30% enhanced the sensory properties of pita bread. Similarly, inclusion of various types of legumes, nuts, and mushroom flour and protein isolates in bread was reported to affect the sensory quality of the products as the supplementation rate elevated [6, 9, 12, 29, 32, 38]. In addition, supplementation of wheat flour with various concentrations of whey protein isolate and buttermilk powder concurrently affected the sensory attributes of the bread [18, 19]. Overall our findings demonstrated that supplementation of bread formula with buttermilk at 30% substitution of added water is recommended for improving the nutritional and sensorial attributes of pan and pita bread.

## 4. Conclusion

The present study focused on the utilization of buttermilk in pan and pita bread making to improve the rheological and nutritional qualities of bread without major effects on the consumer acceptability of the products. The results revealed that incorporation of 30% buttermilk in pan and pita bread formulations significantly improved the rheological properties (water absorption capacity, dough development time, gelatinization temperature, and peak viscosity), physical properties (bread weight, volume, and specific volume), and sensory quality of pan and pita bread. Therefore, supplementation of bread with 30% buttermilk is recommended and could potentially improve the nutritional and sensory qualities of pan and pita breads, those regularly consumed by people in Saudi Arabia, and thus could improve the nutritional and health status of those communities.

## Figures and Tables

**Table 1:** Formulation of pan and pita bread with different concentration of buttermilk.

Ingredients	% of wheat flour	% of added buttermilk			
		0%	30%	60%	100%
*Pan bread*					
Wheat flour (g)	100	1000	1000	1000	1000
^*∗*^Water (mL)	60	600	434	256	—
Buttermilk (mL)	—	—	186	384	675
Yeast (g)	3	30	30	30	30
Sugar (g)	5	50	50	50	50
Salt (g)	2	20	20	20	20
Margarine (g)	3	30	30	30	30
Improver (g)	0.01	0.1	0.1	0.1	0.1
*Pita bread*					
Wheat flour (g)	100	1000	1000	1000	1000
^*∗*^Water (mL)	60	600	434	256	—
Buttermilk (mL)	—	—	186	384	675
Yeast (g)	3	30	30	30	30
Sugar (g)	5	50	50	50	50
Salt (g)	1	10	10	10	10
Improver (g)	0.01	0.1	0.1	0.1	0.1

^*∗*^Added water calculated based on the farinograph results of formulated dough.

**Table 2:** Farinograph readings of wheat dough fortified with different concentrations of buttermilk (30, 60, and 100% of added water).

Dough samples	% of water absorption corrected to 14%	Arrival time (min)	Dough development time (min)	Dough stability (min)	Departure time (min)	Degree of softening (BU)
Control (Wheat flour + 0% butter milk)	60.0	1.5	2.3	9.7	9.7	23.0
Wheat flour + 30% buttermilk	62.0	1.5	2.9	8.3	9.8	22.0
Wheat flour + 60% buttermilk	64.0	1.5	2.7	8.3	9.8	24.0
Wheat flour + 100% buttermilk	67.0	1.5	2.2	8.1	9.6	24.0

Mean values of triplicates, SD ≤ ±5%.

**Table 3:** Amylograph readings of wheat dough fortified with different concentrations of buttermilk (30, 60, and 100%).

Dough samples	Gelatinization temperature (°C)	Peak viscosity (BU)	Temperature at peak viscosity (°C)
Control (wheat flour + 0% buttermilk)	60.7	671.0	88.9
Wheat flour + 30% buttermilk	62.0	781.0	86.9
Wheat flour + 60% buttermilk	62.9	848.0	83.9
Wheat flour + 100% buttermilk	63.0	987.0	82.1

Mean values of triplicates, SD ≤ ±5%.

**Table 4:** Physical characteristics of pan breads made using wheat flour fortified with different concentrations (0, 30, 60, and 100% of added water) of buttermilk.

Physical properties	Fortification rate			
	0%	30%	60%	100%
Loaf weight (g)	483.67 ± 1.08^c^	487.67 ± 1.51^b^	496.00 ± 2.00^a^	498.33 ± 2.30^a^
Loaf volume (cm)	2650.00 ± 12.24^b^	2883.33 ± 28.86^a^	2633.33 ± 17.22^b^	2566.67 ± 10.01^c^
Loaf specific volume (cm^2^/g)	5.48 ± 0.01^b^	5.91 ± 0.07^a^	5.31 ± 0.06^c^	5.15 ± 0.07^d^

^a–d^Mean values of triplicate samples ± SD. Means not sharing a common superscript (s) in a row are significantly different at *P* ≤ 0.05 as assessed by Duncan's Multiple Range Test.

**Table 5:** Chemical composition of pan and pita breads made using wheat flour fortified with different concentrations (0, 30, 60, and 100% of added water) of buttermilk.

Parameters	Fortification rate			
	0%	30%	60%	100%
*Pan bread*				
Protein	13.65 ± 0.30^b^	13.79 ± 0.21^b^	14.21 ± 0.01^a^	14.38 ± 0.23^a^
Oil	4.17 ± 0.13^c^	4.45 ± 0.09^b^	4.55 ± 0.11^b^	4.79 ± 0.05^a^
Ash	2.69 ± 0.10^a^	2.78 ± 0.00^a^	2.75 ± 0.00^a^	2.92 ± 0.01^a^
Carbohydrate	79.53 ± 0.35^a^	79.12 ± 0.10^a^	78.44 ± 0.13^b^	77.89 ± 0.16^c^
*Pita bread*				
Protein	13.41 ± 0.12^b^	13.47 ± 0.39^b^	15.04 ± 0.69^a^	15.17 ± 0.58^a^
Oil	1.54 ± 0.10^c^	2.12 ± 0.06^b^	2.19 ± 0.04^b^	2.98 ± 0.19^a^
Ash	1.71 ± 0.00^b^	1.75 ± 0.05^b^	2.06 ± 0.11^a^	1.98 ± 0.04^a^
Carbohydrate	83.26 ± 0.45^a^	82.29 ± 0.49^a^	80.69 ± 0.34^b^	79.85 ± 0.25^b^

^a–c^Mean values of triplicate samples ± SD. Means not sharing a common superscript (s) in a row are significantly different at *P* ≤ 0.05 as assessed by Duncan's Multiple Range Test.

**Table 6:** Sensory attributes of pan and pita breads made using wheat flour fortified with different concentrations (0, 30, 60, and 100% of added water) of buttermilk.

Parameters	Fortification rate			
	0%	30%	60%	100%
*Pan bread*				
Surface color	9.40 ± 0.29^a^	9.70 ± 0.27^a^	9.30 ± 0.25^a^	9.42 ± 0.37^a^
Degree of symmetry	9.50 ± 0.10^a^	9.60 ± 0.09^a^	9.00 ± 0.17^b^	8.60 ± 0.29^b^
Degree of cracking	9.70 ± 0.38^a^	9.80 ± 0.42^a^	9.10 ± 0.16^b^	8.80 ± 0.28^b^
Crumb color	9.60 ± 0.19^a^	9.50 ± 0.70^a^	9.50 ± 0.30^a^	9.40 ± 0.14^a^
Crumb texture	9.50 ± 0.10^a^	9.60 ± 0.19^a^	9.30 ± 0.12^b^	9.10 ± 0.17^b^
Overall evaluation	47.70 ± 0.54^a^	48.10 ± 1.28^a^	46.20 ± 0.69^b^	45.30 ± 0.70^b^
*Pita bread*				
Surface color	4.80 ± 0.22^a^	4.70 ± 0.18^a^	4.10 ± 0.06^b^	4.60 ± 0.14^a^
Degree of symmetry	4.90 ± 0.31^a^	4.80 ± 0.12^a^	4.30 ± 0.18^b^	4.90 ± 0.01^a^
Degree of cracking	4.70 ± 0.18^a^	4.70 ± 0.22^a^	4.50 ± 0.52^a^	4.80 ± 0.42^a^
Crumb color	5.00 ± 0.05^a^	4.90 ± 0.31^a^	4.30 ± 0.12^b^	4.50 ± 0.12^b^
Crumb texture	4.90 ± 0.31^a^	4.30 ± 0.17^b^	3.80 ± 0.13^c^	4.10 ± 0.11^b^
Taste and flavor	4.80 ± 0.22^a^	4.80 ± 0.12^a^	4.40 ± 0.11^b^	4.10 ± 0.19^b^
Symmetry of top and bottom layers	4.80 ± 0.22^a^	4.60 ± 0.10^a^	4.20 ± 0.13^b^	3.80 ± 0.28^b^
Overall acceptance	4.84 ± 0.18^a^	4.61 ± 0.09^a^	4.22 ± 0.03^b^	4.39 ± 0.08^b^

^a–c^Mean values of triplicate samples ± SD. Means not sharing a common superscript (s) in a row are significantly different at *P* ≤ 0.05 as assessed by Duncan's Multiple Range Test.

## References

[1] Saccotelli M. A., Conte A., Burrafato K. R., Calligaris S., Manzocco L., Del Nobile M. A. (2017). Optimization of durum wheat bread enriched with bran. *Food Science & Nutrition*.

[2] Siebel W., Popper L., Schafer W., Freund W. (2006). *Future of Flour – A compendium of Flour Improvement*.

[3] Aljobair M. O. (2017). Assessment of the Bread Consumption Habits Among the People of Riyadh, Saudi Arabia. *Pakistan Journal of Nutrition*.

[4] Jideani V. A., Onwubali F. C. (2009). Optimisation of wheat-sprouted soybean flour bread using response surface methodology. *African Journal of Biotechnology*.

[5] Iuliana B., Georgeta S., Violeta S., Iuliana A. (2012). Effect of the addition of wheat bran stream on dough rheology and bread quality. *The Annals of the University Dunareade Josof Galati Fascicle VI Food Technology*.

[6] Campbell L., Euston S. R., Ahmed M. A. (2016). Effect of addition of thermally modified cowpea protein on sensory acceptability and textural properties of wheat bread and sponge cake. *Food Chemistry*.

[7] Eissa H. A., Hussein A. S., Mostafa B. E. (2007). Rheological properties and quality evaluation of Egyptian Balady bread and biscuits supplemented with flours of ungerminated and germinated legume seeds or mushroom. *Polish Journal of Food Nutrition Sciences*.

[8] Erukainure O. L., Okafor J. N. C., Ogunji A., Ukazu H., Okafor E. N., Eboagwu I. L. (2016). Bambara–wheat composite flour: rheological behavior of dough and functionality in bread. *Food Science & Nutrition*.

[9] Mohammed I., Ahmed A. R., Senge B. (2012). Dough rheology and bread quality of wheat–chickpea flour blends. *Industrial Crops and Products*.

[10] Ribotta P. D., Arnulphi S. A., León A. E., Añón M. C. (2005). Effect of soybean addition on the rheological properties and breadmaking quality of wheat flour. *Journal of the Science of Food and Agriculture*.

[11] Sabanis D., Tzia C. (2009). Effect of rice, corn and soy flour addition on characteristics of bread produced from different wheat cultivars. *Food and Bioprocess Technology*.

[12] Moktan K., Ojha P. (2016). Quality evaluation of physical properties, antinutritional factors, and antioxidant activity of bread fortified with germinated horse gram (Dolichus uniflorus) flour. *Food Science & Nutrition*.

[13] Conway V., Gauthier S. F., Pouliot Y. (2014). Buttermilk: Much more than a source of milk phospholipids. *Animal Frontiers*.

[14] Kumar R., Kaur M., Garsa A. K., Shrivastava B., Reddy V. P., Tyagi A., Puniya A. K. (2015). Natural and Cultured Buttermilk. *Fermented milk and dairy products*.

[15] Conway V., Couture P., Richard C., Gauthier S. F., Pouliot Y., Lamarche B. (2013). Impact of buttermilk consumption on plasma lipids and surrogate markers of cholesterol homeostasis in men and women. *Nutrition, Metabolism & Cardiovascular Diseases*.

[16] Fuller K. L., Kuhlenschmidt T. B., Kuhlenschmidt M. S., Jiménez-Flores R., Donovan S. M. (2013). Milk fat globule membrane isolated from buttermilk or whey cream and their lipid components inhibit infectivity of rotavirus in vitro. *Journal of Dairy Science*.

[17] Larsson S. C., Andersson S.-O., Johansson J.-E., Wolk A. (2008). Cultured milk, yogurt, and dairy intake in relation to bladder cancer risk in a prospective study of Swedish women and men. *American Journal of Clinical Nutrition*.

[18] Hassan A. A., El-Shazly H. A. M., Sakr A. M., Ragab W. A. (2013). Influence of Substituting Water with Fermented Skim Milk, Acid Cheese Whey or Buttermilk on Dough Properties and Baking Quality of Pan Bread. *World Journal of Dairy Food Science*.

[19] Madenci A. B., Bilgiçli N. (2014). Effect of Whey Protein Concentrate and Buttermilk Powders on Rheological Properties of Dough and Bread Quality. *Journal of Food Quality*.

[20] AACC (2000). *Approved Methods of the AACC*.

[21] Mousa E. I., Al-Mohizea I. S. (1987). Bread baking in Saudi Arabia. *CerealFoodWorld*.

[22] AOAC (2005). *Official Methods of Analysis*.

[23] Hallen E., Ibanoğlu Ş., Ainsworth P. (2004). Effect of fermented/germinated cowpea flour addition on the rheological and baking properties of wheat flour. *Journal of Food Engineering*.

[24] Mashayekh M., Mahmoodi M. R., Entezari M. H. (2008). Effect of fortification of defatted soy flour on sensory and rheological properties of wheat bread. *International Journal of Food Science & Technology*.

[25] Bilgin B., Daðlioðlu O., Konyali M. (2006). Functionality of bread made with pasteurized whey and /or buttermilk. *Italian Journal of Food Science*.

[26] Anton A. A., Ross K. A., Lukow O. M., Fulcher R. G., Arntfield S. D. (2008). Influence of added bean flour (Phaseolus vulgaris L.) on some physical and nutritional properties of wheat flour tortillas. *Food Chemistry*.

[27] Gadallah M. G. E., Rizk I. R. S., Elsheshetawy H. E., Bedeir S. H., Abouelazm A. M. (2017). Impact of Partial Replacement of Wheat Flour with Sorghum or Chickpea Flours on Rheological Properties of Composite Blends. *Journal of Agricultural and Veterinary Sciences*.

[28] Pasha I., Rashid S., Anjum F. M., Sultan M. T., Nasir Qayyum M. M., Saeed F. (2011). Quality evaluation of wheat-mungbean flour blends and their utilization in baked products. *Pakistan Journal of Nutrition*.

[29] Majeed M., Khan M. U., Owaid M. N. (2017). Development of oyster mushroom powder and its effects on physicochemical and rheological properties of bakery products. *Journal of Microbiology, Biotechnology and Food Sciences*.

[30] Adebowale A. A., Adegoke M. T., Sanni S. A., Adegunwa M. O., Fetuga G. O. (2012). Functional properties and biscuit making potentials of sorghum-wheat flour composite. *American Journal of Food Technology*.

[31] Hrušková M., Švec I., Jurinová I. (2013). Chemometrics of wheat composites with hemp, teff, and chia flour: Comparison of rheological features. *International Journal of Food Science*.

[32] Pejcz E., Mularczyk A., Gil Z. (2015). Technological characteristics of wheat and non-cereal flour blends and their applicability in bread making. *Journal of Food and Nutrition Research*.

[33] Zaidul L. S., Abd Karim A., Manan D. M. A., Nik Norulaini N. A., Omar A. K. M. (2003). Gelatinization Properties of Sago and Wheat Flour Mixtures. *ASFAN Food Journal*.

[34] Gani A., Broadway A. A., Masoodi F. A. (2015). Enzymatic hydrolysis of whey and casein protein- effect on functional, rheological, textural and sensory properties of breads. *Journal of Food Science and Technology*.

[35] Wani A. A., Sogi D. S., Singh P., Sharma P., Pangal A. (2012). Dough-handling and cookie-making properties of wheat flour-watermelon protein isolate blends. *Food and Bioprocess Technology*.

[36] Lin L.-Y., Liu H.-M., Yu Y.-W., Lin S.-D., Mau J.-L. (2009). Quality and antioxidant property of buckwheat enhanced wheat bread. *Food Chemistry*.

[37] Madenci B., Turker S., Bilgicli N. Effect of some dairy by-product on physical, chemical and sensory properties of lavash bread.

[38] Ndife J., Abdulraheem L. O., Zakari U. M. (2011). Evaluation of the nutritional and sensory quality of functional breads produced from whole wheat and soya bean flour blends. *African Journal of Food Science*.

